# Draft Genome Sequence of *Pantoea agglomerans* JM1, a Strain Isolated from Soil Polluted by Industrial Production of Beta-Lactam Antibiotics That Exhibits Valacyclovir-Like Hydrolase Activity

**DOI:** 10.1128/genomeA.00921-17

**Published:** 2017-09-21

**Authors:** Jiří Zahradník, Martina Plačková, Andrea Palyzová, Helena Marešová, Eva Kyslíková, Pavel Kyslík

**Affiliations:** a Institute of Microbiology, Czech Academy of Sciences, v.v.i., Prague, Czech Republic; b Faculty of Science, Charles University Prague, Prague, Czech Republic

## Abstract

Strain *Pantoea agglomerans* JM1 was isolated from the soil of a microbiome that had been exposed to polluting pharmaceuticals. The bacterium exhibited enzymatic activities useful for the biotransformation of beta-lactams. The genome of the strain was assembled and described, and the gene encoding valacyclovir-like hydrolase was identified.

## GENOME ANNOUNCEMENT

The microorganism *Pantoea agglomerans* is a Gram-negative motile aerobic enterobacterium (1). So far, 25 species belonging to this genus have been recognized (2). Members of the genus *Pantoea* have a broad ability to colonize different environments and have been found in association with plants (3, 4), marine sediments (5), a hypersaline lake (6), and clinical samples (7, 8). Some strains of *P. agglomerans* are commercially available as biocontrol agents that reduce the incidence of basal kernel blight of barley, fire blight, and other diseases mainly caused by the pathogenic bacteria *Pseudomonas siringae* pv. syringae (9) and *Erwinia amylovora* (10, 11), as well as fungal pathogens (12).

The strain *Pantoea agglomerans* JM1 (Czech Collection of Microorganisms, CCM 8766) was isolated by extensive screening of microorganisms from a soil sample polluted by a factory producing beta-lactam antibiotics (Slovenská L’upča). The aim of the study was to identify new enzymes useful for the biotransformation of beta-lactams, with activities analogous to alpha-amino acid ester hydrolases (AEHs) (13) or penicillin G acylases (PGAs) (14, 15). The enzymes were detected by the hydrolysis of a chromogenic substrate, 6-nitro-3-(phenylacetamido)benzoic acid (NIPAB) (16, 17). The strain JM1 showed a weak-positive phenotype for NIPAB hydrolysis. Surprisingly, no genes encoding AEHs and PGAs were determined by PCR and subsequently by genome data. The existence of novel enzymes hydrolyzing NIPAB may have a great biotechnological potential. Therefore, we decided to identify the enzyme with this activity in the genome. Predicted genes were cloned and expressed in *Escherichia coli* BL21. The protein-hydrolyzing NIPAB had an alpha/beta hydrolase fold (WP_069025498) remotely homologous (22%) to human valacyclovirase.

Total genomic DNA was isolated as described previously (18) and sequenced on the Illumina HiSeq 2000 platform, with a paired-end approach, by Beijing Genomics Institute. A total of 7,036,164 reads (868 Mb of clean data) were assembled with SOAPdenovo version 2.04 and SOAPaligner version 2.21 using the complete genome of *Pantoea* sp. At-9b (GenBank accession no. GCF_000175935.2) as a reference; 210,790 single nucleotide polymorphisms in corresponding regions were found. The genome size of *Pantoea agglomerans* JM1 was 4,794,872 bp in 34 scaffolds with an overall G+C content 55.14%. We predicted 4,728 genes with BASys (19), 8 rRNAs with the WebMGA prediction server (20), and 71 tRNAs (3 pseudo) with tRNAscan-SE (21). Plasmids pPag1 and pPag3 (unlocalized in one scaffold) were identified as being closely related to those of *Pantoea vagans* C9-1 (22, 23).

Multilocus sequence analysis based on *atpD*, *glnA*, *gyrB*, and *recA* showed a >98% similarity with *Pantoea agglomerans* C410P1 and confirmed the phylogenetic position of the species. The average nucleotide identity using the BLAST (ANIb) value for the closely related strain *Pantoea agglomerans* MP2 is 98.63%, as computed with JSpeciesWS software (24).

The draft genome sequence of *Pantoea agglomerans* JM1 presented here is a remarkable source of information on genes encoding novel enzymes with industrial potential. Regarding environmental pollution, the genome represents the key to understanding microbial adaptation to the permanent pressure of industrial pollutants.

### Accession number(s).

The results obtained from this whole-genome shotgun project have been deposited at DDBJ/EMBL/GenBank under the accession no. NHAS00000000.
